# The Differential Effects of HDL Subpopulations on Lipoprotein Lipase (LPL)-Mediated VLDL Catabolism

**DOI:** 10.3390/biomedicines9121839

**Published:** 2021-12-05

**Authors:** Ewa Wieczorek, Agnieszka Ćwiklińska, Agnieszka Kuchta, Barbara Kortas-Stempak, Anna Gliwińska, Maciej Jankowski

**Affiliations:** Department of Clinical Chemistry, Faculty of Pharmacy, Medical University of Gdańsk, 80-211 Gdańsk, Poland; agnieszka.cwiklinska@gumed.edu.pl (A.Ć.); agnieszka.kuchta@gumed.edu.pl (A.K.); barbara.kortas-stempak@gumed.edu.pl (B.K.-S.); anna.gliwinska@gumed.edu.pl (A.G.); maciej.jankowski@gumed.edu.pl (M.J.)

**Keywords:** apolipoprotein C, apolipoprotein E, high-density lipoprotein, HDL-2, HDL-3, lipolysis, very-low-density lipoprotein

## Abstract

High-density lipoprotein (HDL) subpopulations functional assessment is more relevant for HDL anti-atherogenic activity than cholesterol level. The aim of the study was to assess the impact of HDL-2 and HDL-3 on lipoprotein lipase (LPL)-mediated very-low-density lipoprotein (VLDL) catabolism related to hypertriglyceridemia development. VLDL and HDLs were isolated from serum by ultracentrifugation. VLDL was incubated with LPL in the absence and presence of total HDL or HDL subpopulations. Next, VLDL remnants were separated, and their composition and electrophoretic mobility was assessed. Both HDL subpopulations increased the efficiency of triglyceride lipolysis and apolipoprotein CII and CIII removal from VLDL up to ~90%. HDL-3 exerted significantly greater impact than HDL-2 on apolipoprotein E (43% vs. 18%, *p* < 0.001), free cholesterol (26% vs. 18%, *p* < 0.05) and phospholipids (53% vs. 43%, *p* < 0.05) removal from VLDL and VLDL remnant electrophoretic mobility (0.18 vs. 0.20, *p* < 0.01). A greater release of these components was also observed in the presence of total HDL with a low HDL-2/HDL-3 cholesterol ratio. Both HDL subpopulations affect VLDL composition during lipolysis, but HDL-3 exhibited a greater effect on this process. Altered composition of HDL related to significant changes in the distribution between HDL-2 and HDL-3 can influence the VLDL remnant features, affecting atherosclerosis progression.

## 1. Introduction

Atherosclerotic cardiovascular disease (ASCVD) is one of the leading causes of mortality in the world, accounting for approximately 30% of global deaths, and disturbances in lipid metabolism are one of the key factors leading to ASCVD development [1]. A low concentration of high-density lipoprotein cholesterol (HDL-C) is traditionally related to a higher risk of suffering cardiovascular events; however, HDL-C-targeted therapy has failed to reduce these events [2]. Therefore, it is now considered that HDL functionality, rather than HDL-C level, can better reflect the anti-atherogenic effect and protective role of these lipoproteins [3]. HDL is a very heterogeneous population of particles and it has been shown that the HDL subpopulation distribution can be a better predictor of cardiovascular disease (CVD) risk [4]. Hence, increasing emphasis is now placed on studying HDL subpopulations and the functionality of these particles rather than on the concentration of HDL-C [1].

HDL can be divided into several subpopulations depending on their size, apolipoprotein content, mass and charge or density [5]. The separation by density allows HDL to be split into two main subpopulations: smaller, protein-enriched HDL-3 particles with a density of 1.125–1.21 g/mL and larger HDL-2 particles with a lower than for HDL-3 protein:lipid ratio and density of 1.063–1.125 g/mL. HDL-3 is continuously converted into HDL-2 and vice versa by a multifactorial process of HDL remodeling which uses several enzymes and transfer proteins and which also affects the metabolism of other lipoproteins. The data on the antiatherosclerotic effects of the HDL subpopulations is not consistent and has changed over time, but HDL-3 is now considered to possibly exert more a favorable antiatherosclerotic effect than HDL-2 [2,4]. 

HDL has a large variety of anti-atherogenic functions, including participation in reverse cholesterol transport (RCT), as well as anti-oxidative, anti-inflammatory, anti-thrombotic and anti-apoptotic activities [6]. It also has an important role in the intravascular metabolism of triglyceride (TG)-rich lipoproteins, such as chylomicrons and very-low-density lipoprotein (VLDL) [7]. A key pathway linking plasma HDL and VLDL metabolism can be manifested as a negative correlation between HDL-C and TG plasma levels [8]. It includes cholesteryl ester transfer protein (CETP)-mediated heteroexchange of TG and cholesteryl esters (CE) between the lipoproteins, as well as acquisition by HDL of the surface components released from VLDL during lipoprotein lipase (LPL)-mediated TG lipolysis [7]. This acquisition is also required for HDL maturation and is considered a quantitatively major source of circulating HDL-C [7]. 

The removal of surface components allows VLDL to be efficiently converted into smaller remnant particles, which can then be taken up by liver receptors or further metabolized to low-density lipoprotein (LDL) [9]. The surface components released from VLDL during lipolysis consist of lipids: free cholesterol (FC) and phospholipids (PL), and exchangeable apolipoproteins (apo): apo CII, apo CIII and apo E. The apolipoproteins influence LPL activity and participate in VLDL catabolism. Apo CII is an activator of LPL [10], whereas apo CIII acts as an LPL inhibitor [11]. Apo E has a dual role in VLDL catabolism, as it promotes hepatic clearance of VLDL and their remnants, but at high levels, it can impair LPL activity by displacing the LPL activator, apo CII, from the particles [12]. 

Impaired catabolism of VLDL is one of the main causes of hypertriglyceridemia (HTG) development and the decreased efficacy of TG lipolysis is crucial to this process [13]. Delayed TG hydrolysis prolongs the retention time of remnant particles and, consequently, increases their amounts in the blood vessels. The remnants particles, due to their smaller diameters, penetrate the blood vessel endothelium more easily than VLDL itself and accumulate in the vessels, thereby promoting the acceleration of atherosclerotic plaque formation [14]. The increased amounts of remnants enhance the expression of adhesion molecules, apoptosis of endothelial cells, platelet activation, smooth muscle cell proliferation and intensify the local inflammatory process [14]. Consequently, the accumulation of remnant particles significantly accelerates the development of atherosclerosis [14]. 

The acquisition of VLDL surface components by HDL during LPL-mediated lipolysis is the basis of the reverse-remnant cholesterol transport (RRT) hypothesis, which considers that an impaired transfer of cholesterol from TG-rich lipoproteins to HDL upon lipolysis plays an important role in the development of CVD and may result from a decreased level or altered composition of HDL particles [7]. Therefore, the aim of our study was to evaluate the impact of HDL-2 and HDL-3 subpopulations on LPL-mediated VLDL lipolysis efficiency and on the magnitude of the release of surface components from VLDL. We found that although HDL-2 and HDL-3 had comparable effects on VLDL-TG lipolysis efficiency, they differed in their ability to release surface components from VLDL, thereby resulting in the generation of VLDL remnants with different properties.

## 2. Materials and Methods

### 2.1. Materials

Venous blood was collected after overnight fasting into commercially available test tubes (BD Vacutainer, Franklin Lakes, NJ, USA) and centrifuged to obtain the serum.

Blood was collected from adult volunteers (n = 10, age: 44 ± 15 years, 60% female) who were asymptomatic for CVD at the time of recruitment and had no history of previous cardiovascular events; all were under the care of the General Practitioner (GP) (Non-public Health Care Centre, Pomerania region, Poland). The study exclusion criteria were acute diseases within 3 months before the study, diseases affecting lipid metabolism (liver diseases, kidney diseases, diabetes), active phase of a cancer disease, treatment with lipid lowering drugs (statins, fibrates, ezetimib, and PCSK9 inhibitors), heparin or other drugs affecting the lipid profile (steroids, diuretics and immunosuppressive agents). The lipid parameters of the study group were as follows: TG–152 ± 61 mg/dL, total cholesterol–222 ± 37 mg/dL, LDL cholesterol–137 ± 32 mg/dL.

The study was approved by the Independent Bioethics Commission for Research of the Medical University of Gdańsk, Poland (No. NKBBN/612/2017–2018, date 23 January 2018). All participants gave written informed consent for participation in the study and for publication of the results of this research.

### 2.2. Lipoprotein Isolation

VLDL was isolated from sera by ultracentrifugation according to the procedure described earlier [15]. Total HDL (HDL_T_) and the HDL subpopulations: HDL-2 and HDL-3 were isolated from the infranatant after VLDL isolation by combining precipitation method and ultracentrifugation procedure described by McPherson [16], with some modification. Briefly, the apo B-containing lipoproteins remaining in the infranatant after VLDL isolation were precipitated by heparin (5000 U/mL) (Polfa Warszawa, Warsaw, Poland) and 1M MnCl_2_. Next, the HDL was adjusted to a density of 1.21 g/mL by adding solid KBr, transferred into a 3.2 mL ultracentrifuge tube (Polyallomer Bell-top, Beckman Coulter, Brea, CA, USA) and respective volumes of an aqueous solution of KBr with a density of 1.21 g/mL was added. Ultracentrifugation was performed for 150 min at 541,000× *g* and 4 °C in a Beckman Optima^TM^ TLX Ultracentrifuge using a Beckman fixed-angle rotor (TLA 100.3). After ultracentrifugation, 1.9 mL of supernatant containing HDL_T_ (HDL-2+HDL-3) was collected by tube slicing (Beckman Tube Slicer, Beckman Coulter, Brea, CA, USA). 

To isolate HDL-2 and HDL-3 subpopulations, HDL obtained after apoB-containing lipoprotein precipitation with heparin and MnCl_2_ was adjusted to a density of 1.125 g/mL by adding solid KBr, transferred into a 3.2 mL ultracentrifuge tube (Polyallomer Bell-top, Beckman Coulter, Brea, CA, USA) and respective volumes of an aqueous solution of KBr with a density of 1.125 g/mL was added. Ultracentrifugation was then performed under the conditions described above, and 1.9 mL of supernatant containing HDL-2 was collected by tube slicing. The remaining infranatant was adjusted to a density of 1.21 g/mL by adding solid KBr, transferred into a 3.2 mL ultracentrifuge tube (Polyallomer Bell-top, Beckman Coulter, Brea, CA, USA), refilled with respective volumes of an aqueous solution of KBr with a density of 1.21 g/mL and ultracentrifuged under the conditions described above. After ultracentrifugation, 1.9 mL of supernatant containing HDL-3 was collected by tube slicing (Beckamn Tube Slicer, Beckman Coulter, Brea, CA, USA). 

The VLDL, HDL_T,_ HDL-2 and HDL-3 subpopulations were dialyzed against 0.01 M Tris-HCl buffer, pH 7.4, containing 0.195 M NaCl and 0.01% NaN_3_ as a preservative for 18 h, 4 °C [17]. The obtained lipoproteins were characterized by their lipids: total cholesterol (TC), free cholesterol (FC), triglyceride (TG) and phospholipid (PL), and apolipoproteins: apo AI, apo B apo CII, apo CIII, and apo E composition, and used in the VLDL lipolysis study. The composition of lipoproteins used in the study is presented in Table 1.

### 2.3. VLDL Lipolysis Study

VLDL was incubated for 1 h at 37 °C with LPL isolated from bovine milk (Sigma Aldrich, St. Louis, MO, USA) at a constant VLDL-TG:LPL 90:0.48 mg/dL ratio [17] in the absence or presence of HDL_T_ or HDL subpopulations: HDL-2 and HDL-3. The weight ratio of VLDL cholesterol:HDL cholesterol (VLDL-C:HDL-C) was 1:1, regardless of the HDL subpopulation used, and albumin (Sigma Aldrich, St. Louis, MO, USA) was added to the reaction mixtures as a free fatty acids acceptor (final concentration in reaction mixture: 2% *w*/*w*) [17]. Volume differences between reaction mixtures were corrected by adding an appropriate volume of 0.01 M Tris-HCl buffer, pH 7.4, containing 0.195 M NaCl and 0.01% NaN_3_ corresponding to the volume of HDL and/or LPL added. The control reaction mixture contained VLDL, albumin and the incubation buffer (described above). 

After incubation, the mixtures were cooled on ice for 10 min. Next, to assess the electrophoretic mobility of the VLDL remnants agarose gel electrophoresis was performed. The VLDL remnant composition was evaluated by separating the remnants from other reaction products by immunoprecipitation with anti-apo B polyclonal antibodies (Dako, Glostrup, Denmark), as described previously [18], or by ultracentrifugation. Briefly, 0.5 mL of each reaction mixture was adjusted to a density of 1.063 g/ mL by adding solid KBr. The density-adjusted reaction mixture was transferred into a 1.5 mL ultracentrifuge tube (Polyallomer Bell-top, Beckman Coulter, Brea, CA, USA) and respective volume of an aqueous solution of KBr (density 1.063 g/mL) was added. Samples were ultracentrifuged in a Beckman OptimaTM TLX Ultracentrifuge using a Beckman fixed-angle rotor (TLA 120.2) for 180 min, at 541,000× *g*, 4 °C, acceleration 5, deceleration 9. After ultracentrifugation, 0.35 mL of supernatant containing VLDL remnants was collected. 

The lipid components (FC, TG, and PL) and exchangeable apolipoproteins (apo CII, apo CIII, and apo E) were measured in the isolated VLDL remnants and a VLDL control. The percentage of hydrolyzed TG and the percentage of lipids and apolipoproteins released from VLDL during lipolysis were established by comparing the component concentrations in the remnants after lipolysis to those in the control VLDL. 

### 2.4. Biochemical Analysis and Agarose Electrophoresis

The concentration of lipid components was measured using commercially available enzymatic kits obtained from Pointe Scientific, Warsaw, Poland (TG, TC), DiaSys, Holzheim, Germany (FC), and Wako Diagnostics, Mountain View, CA, USA (PL). The apolipoprotein concentration was measured by immunonephelometry using kits obtained from Siemens Healthcare, Erlangen, Germany (apo AI, apo B, apo E) and Randox, Warsaw, Poland (apo CII, apo CIII). 

Agarose electrophoresis of lipoproteins was performed for 1.5 h using a commercially available Hydragel 7 LIPO + Lp(a) kit (Sebia, Lisses, France). Electropherograms were stained with Sudan Black and analyzed by densitometry with Launch VisionWorksLS 7.0.1 software (Thermo Fisher Scientific, Waltham, MA, USA). The relative electrophoretic mobility of the samples was calculated by dividing the distance of the lipoprotein band measured from the sample application site by the distance of the dye front measured from the sample application site. 

### 2.5. Statistical Analysis

Statistical analysis was performed using GraphPad Prism 5 software (GraphPad Software, San Diego, CA, USA). The normality of distribution of continuous variables or differences was assessed with the Shapiro-Wilk normality test. Data were presented as mean ± standard deviation (mean ± SD) or as median with 25th and 75th percentiles, if appropriate. The difference between the two groups was assessed by Paired *t*-test, Wilcoxon matched pairs test, or the Unpaired *t*-test, if appropriate. The difference between more than two groups was assessed by Repeated Measures ANOVA with a Tukey post-hoc test. Values of *p* < 0.05 were set as statistically significant. 

## 3. Results

### 3.1. The Impact of HDL Subpopulations on VLDL-TG Lipolysis Efficiency

**Figure 1:**
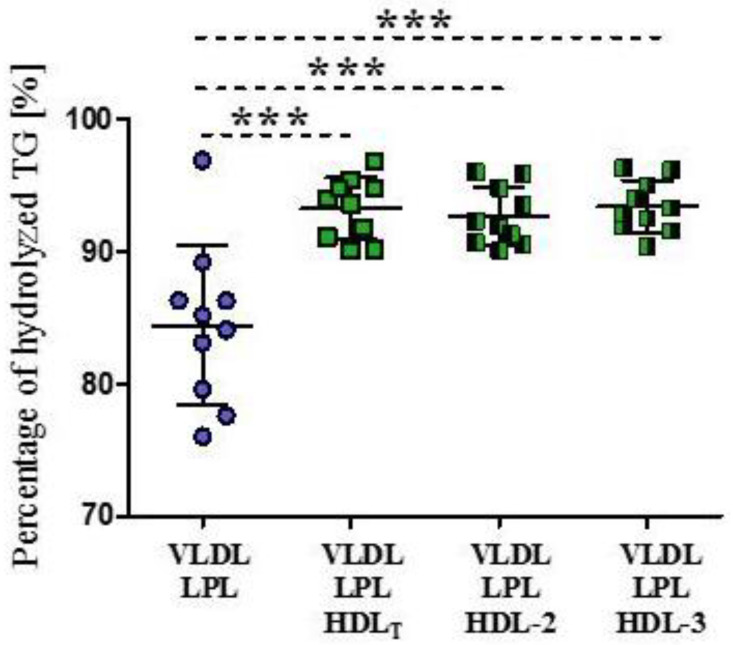
Percentage of hydrolyzed TG during LPL-mediated VLDL lipolysis in the absence and presence of HDL_T_ and HDL subpopulations: HDL-2 and HDL-3. Data are presented as mean ± SD, n = 10, *** *p* < 0.001. Data were analyzed by Repeated Measures ANOVA with Tukey post hoc.

In the absence of HDL, the mean percentage of hydrolyzed VLDL-TG was 84 ± 6%. In the presence of HDL_T_ and HDL subpopulations, the mean percentage of hydrolyzed VLDL-TG was higher by 9% (*p* < 0.001), on average, with no significant difference between the percentage of VLDL-TG hydrolyzed in the presence of HDL-2 and HDL-3 (Figure 1).

### 3.2. Impact of HDL Subpopulations on the Release of Surface Lipids from VLDL during Lipolysis

**Figure 2:**
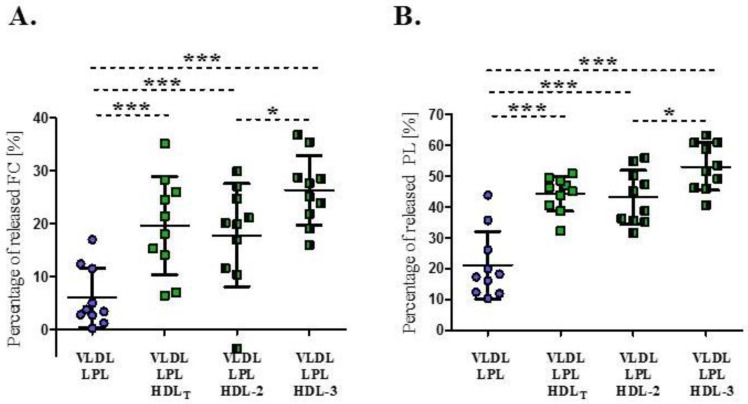
Percentage of free cholesterol (**A**) and phospholipids (**B**) released from VLDL during LPL-mediated lipolysis in the absence or presence of HDL_T_ and HDL subpopulations: HDL-2 and HDL-3. Data are presented as mean ± SD, n = 10, * *p* < 0.05; *** *p* < 0.001. Data were analysed by Repeated Measures ANOVA with Tukey post hoc.

LPL-mediated lipolysis in the absence of HDL led to an average decrease in FC and PL content in VLDL of 6 ± 5.6% and 21 ± 11%, respectively (Figure 2). The degree of surface lipid released from VLDL varied between individuals, ranging from 0.2% to 17% for FC and from 10% to 44% for PL. 

The average percentages of released surface lipids in the presence of HDL_T_, HDL-2 or HDL-3 were 20 ± 9%, 18 ± 10% and 26 ± 7% for FC and 44 ± 6%, 43 ± 8% and 53 ± 8% for PL, respectively, and they were significantly higher compared to result obtained in the absence of HDL (*p* < 0.001) (Figure 2). HDL-3 exerted a greater impact on the release of FC and PL from VLDL during lipolysis, compared to the results obtained in the presence of HDL-2 (*p* < 0.05) (Figure 2).

### 3.3. Impact of HDL Subpopulations on the Release of Exchangeable Apolipoproteins from VLDL during Lipolysis

**Figure 3:**
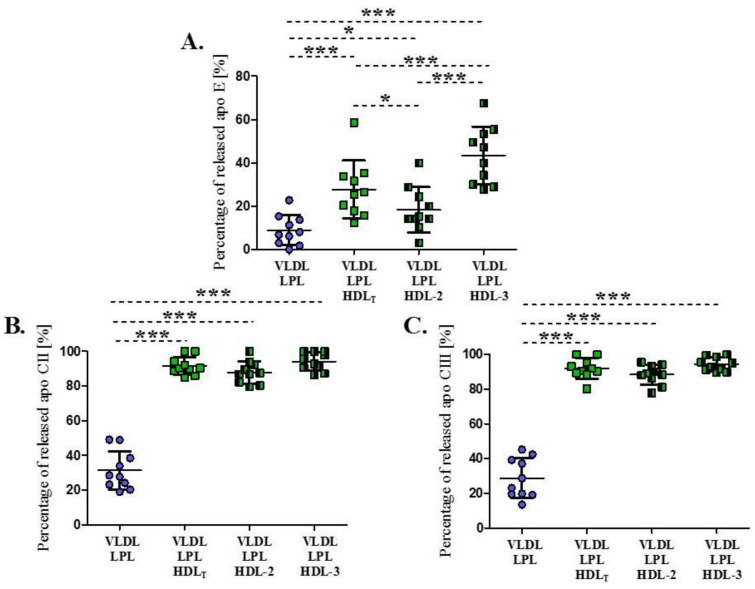
Percentage of apo E (**A**), apo CII (**B**) and apo CIII (**C**) released from VLDL during LPL-mediated lipolysis in the absence or presence of HDL_T_ and HDL subpopulations: HDL-2 and HDL 3. Data are presented as mean ± SD, n = 10, * *p* < 0.05; *** *p* < 0.001. Data were analyzed by Repeated Measures ANOVA with Tukey post hoc.

The concentration of exchangeable apolipoproteins (apo E, apo CII, apo CIII) in VLDL decreased after lipolysis in the absence and presence of HDL (Figure 3). In the absence of HDL, the decrease in VLDL apo E, apo CII and apo CIII content were on average by 9 ± 7%, 31 ± 11%, and 29 ± 11%, respectively (Figure 3). The decrease in VLDL apolipoprotein content differed between individual experiments, ranging from 0% to 23% for apo E, from 19% to 49% for apo CII and from 14% to 45% for apo CIII. 

The mean percentage of apo E released from VLDL was significantly higher in the presence of HDL than in the absence of HDL and a significant difference was observed for the release rate between the HDL subpopulations. The highest average percentage (43 ± 13%) was obtained for HDL-3 and the lowest (18 ± 10%) for HDL-2 (Figure 3A). HDL_T_ had an intermediate effect on apo E release from VLDL; the percentage was 10% higher, on average, compared to the values obtained in the presence of HDL-2 (*p* < 0.05), and 15% lower compared to the values in the presence of HDL-3 (*p* < 0.001) (Figure 3A). 

The mean percentages of apo CII and apo CIII released from VLDL in the presence of HDL were significantly higher (*p* < 0.001), compared to apo Cs released during lipolysis in the absence of HDL. For both apo CII and apo CIII, the release averaged about 88% and 94% in the presence of HDL-2 and HDL-3, respectively, and there was no significant difference for the apo Cs release rates between the HDL subpopulations (Figure 3B,C).

### 3.4. Electrophoretic Mobility of VLDL Remnants Produced during Lipolysis in the Absence and Presence of HDL Subpopulations

**Figure 4:**
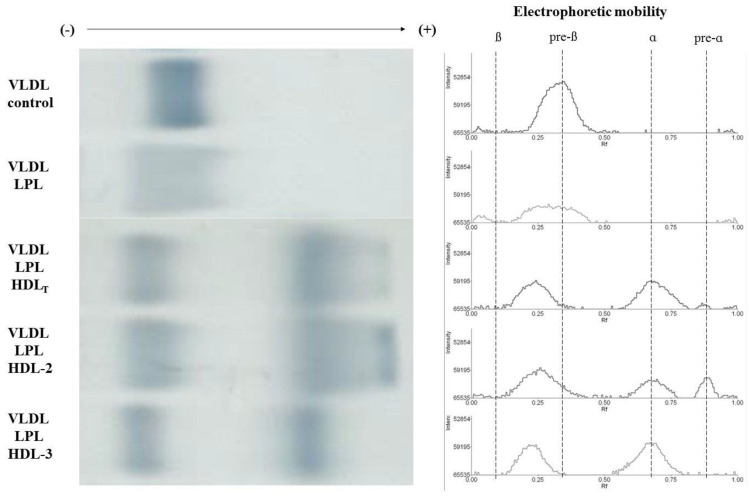
Exemplary electrophoresis and electropherogram of VLDL control and VLDL remnants produced during LPL-mediated lipolysis in the absence or presence of HDL_T_ and HDL subpopulations: HDL-2 and HDL-3; ß mobility corresponds to LDL, pre-ß mobility corresponds to VLDL, ɑ mobility corresponds to HDL.

**Figure 5:**
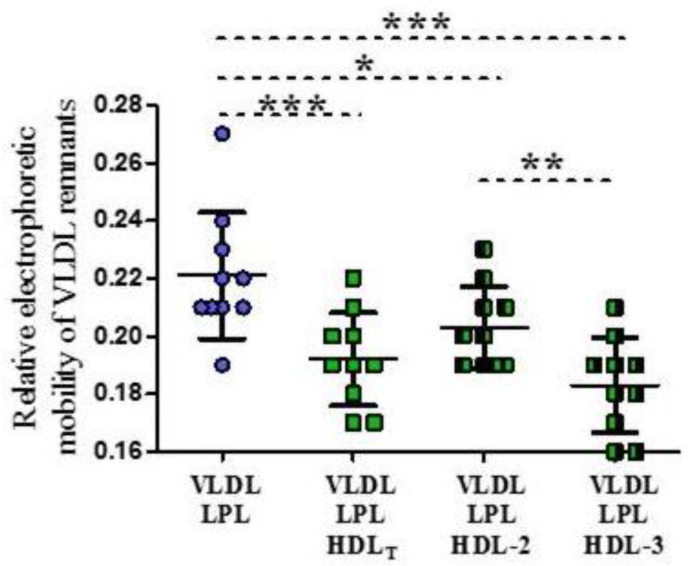
Relative electrophoretic mobility of VLDL remnants produced during LPL-mediated lipolysis in the absence or presence of HDL_T_ and HDL subpopulations: HDL 2 and HDL-3. Data are presented as mean ± SD, n = 10, * *p* < 0.05; ** *p* < 0.01; *** *p* < 0.001. Data were analyzed by Repeated Measures ANOVA with Tukey post hoc.

The mean relative electrophoretic mobility of the VLDL control was 0.30 ± 0.02, whereas the VLDL remnants produced in the absence or presence of HDL had significantly lower relative electrophoretic mobility compared to the control (*p* < 0.001). The remnants produced in the presence of HDL_T_ and HDL subpopulations had lower electrophoretic mobility, compared to remnants produced in the absence of HDL (Figure 4 and Figure 5). Additionally, the remnants produced in the presence of HDL-3 had significantly lower mobility than the remnants produced in the presence of HDL-2 (*p* < 0.01) (Figure 5). 

In the presence of HDL_T_ and HDL-2 the formation of particles with pre-alpha mobility was observed, that were not formed in the presence of HDL-3 (Figure 4). Particles with pre-alpha mobility produced in the presence of HDL-2 had greater intensity on electropherograms than those produced in the presence of HDL_T_ (Figure 4). 

### 3.5. Impact of the HDL-2/HDL-3 Cholesterol Ratio in HDL_T_ on the Release of Lipids and Apolipoproteins from VLDL during Lipolysis

Significantly higher percentages of surface lipids and apo E and apo CII were released from VLDL during lipolysis in the presence of HDL_T_ with a low HDL-2/HDL-3 cholesterol ratio (values below the median of 1.56 calculated for the study group) than in the presence of HDL_T_ with an HDL-2/HDL-3 cholesterol ratio above 1.56 (Table 2). The greatest differences between the groups with low and high HDL-2/HDL-3 cholesterol ratios were observed for FC (2.2-fold) and apo E (1.8-fold). 

## 4. Discussion

In the present study, we assessed the impact of HDL-2 and HDL-3 on LPL-mediated VLDL-TG lipolysis and surface material release from VLDL during lipolysis. We found that although both HDL subpopulations increased VLDL-TG lipolysis efficiency and the removal of surface material from VLDL, HDL-3 exhibited greater impact on this process and promoted a more efficient release of surface components, especially apo E and FC, thereby affecting the properties of the generated remnants. 

Previous in vitro studies have shown that VLDL lipolysis occurs in the absence and presence of HDL, and that HDL increases VLDL-TG lipolysis efficiency in a concentration-dependent manner [17,19]. The release of surface material from VLDL that accompanies lipolysis is necessary for the conversion of VLDL into VLDL remnants and, subsequently, into LDL [7]. HDL plays an important role in this process, as it is an efficient acceptor of excess surface components removed from VLDL since these components cannot be incorporated into the remnant lipoprotein structure possessing reduced hydrophobic core [20,21]. 

The results of our study are consistent with the others as we observed that HDL_T_ significantly increased the VLDL-TG lipolysis efficiency, as well as the release of surface lipids (FC, PL) and exchangeable apolipoproteins (apo E, apo Cs) from VLDL [20,21,22,23]. Moreover, we observed that both HDL subpopulations, HDL-2 and HDL-3, had a similar beneficial effect on the VLDL-TG lipolysis efficiency. Regardless of the used HDL subpopulation, the percentage of hydrolyzed TG was high, at above 90%. Taskinen et al. also reported that the degree of lipolyzed VLDL-TG was comparable regardless of whether the HDL-2 or HDL-3 subpopulation was used. However, they did not observe the effect of the presence of HDL on the effectiveness of VLDL lipolysis compared to the lipolysis without HDL [24]. 

Conversely, analyzing the impact of HDL subpopulations on surface lipids release from VLDL we found that HDL-3 exerted a greater effect on FC and PL removal, compared to HDL-2. Feng et al. recently reported that HDL-3 was about a 3.8-fold more efficient acceptor for FC than large, light HDL-2 [20] while for PL removal they observed a tendency to be more efficient in the presence of the small, dense HDL-3 than in the presence of HDL-2, but the difference was not statistically significant [22].

Regarding the release of exchangeable apolipoproteins, we found that lipolysis in the absence of HDL resulted in a loss of approximately 30% of the baseline of apo Cs content from VLDL, whereas in the presence of HDL it was ~three-fold higher. Glangeaud-Freudenthal et al. also showed that the percentage of released apo CII and apo CIII from VLDL was higher in the presence of HDL and that the released apo Cs was recovered mainly in a subpopulation with a density of about 1.10 g/mL corresponding to the density of HDL-2 [25]. Earlier studies also demonstrated an almost complete loss of apo CII and apo CIII from VLDL in the presence of HDL reaching a value of 90-100% [23,26]. Eisenberg et al. showed that neither apo CII nor apo CIII was preferentially removed from VLDL and that both apolipoproteins were released to the same extent [27]. We also observed a similar release rate for apo CII and apo CIII from VLDL. Moreover, there was no difference between the percentage of released apo Cs in the presence of HDL_T_, HDL-2 and HDL-3 in our study. Yamazaki et al. demonstrated that during the interaction between VLDL and HDL subpopulations but with no LPL, apo CII and apo CIII were transferred to both, HDL-2 and a larger fraction of small HDL-3 [28]. In our previous study, we also observed a similar transfer of apo CII and apo CIII to phosphatidylcholine liposomes in both the presence and absence of LPL [29]. These findings can indicate that the presence of an acceptor is the main factor affecting the efficiency of the release of apo CII and apo CIII from VLDL. Loss of apo Cs from VLDL during lipolysis is important for further apo B-containing lipoprotein metabolism because both apo CII [10] and apo CIII [11] inhibit the binding of these lipoproteins to the LDL receptor (LDLR) and consequently suppress their further hepatic catabolism.

Compared to apo Cs, the percentage of apo E released from VLDL during lipolysis was much lower and amounted on average 9% and 28% in the absence or presence of HDL_T_ respectively. It should be emphasized that the type of HDL acceptor particles had a significant effect on the size of the released apo E pool and the greatest decrease in apo E content in VLDL (1.6-fold and 2.4-fold higher compared to HDL_T_ and HDL-2, respectively) was observed for VLDL incubated with HDL-3. This observation was confirmed by the results obtained in the presence of HDL_T_ analyzed according to the HDL-2/HDL-3 cholesterol ratio. The apo E decrease in VLDL was significantly higher in the presence of HDL_T_ with a low HDL-2/HDL-3 cholesterol ratio than for samples with a high ratio. A similar significant difference (2.2-fold) was also observed for FC release. The data on the impact of HDL subpopulation on apo E release from VLDL upon LPL-mediated lipolysis are scarce. Yamazaki et al., analyzing the interaction between VLDL and HDL but without LPL, found that although apo E released from VLDL was transported to both HDL subpopulations, it was preferentially found in the larger fraction of small HDL-3 particles [28]. 

The differences in VLDL lipolysis efficiency and surface components release in the absence and presence of HDL are in agreement with the differences observed for remnants relative electrophoretic mobility since VLDL remnants produced in the presence of HDL had lower electrophoretic mobility than those produced in the absence of HDL. Moreover, the lowest relative electrophoretic mobility obtained for remnants produced in the presence of HDL-3 (similar to the beta characteristic for LDL) can indicate a greater impact of HDL-3 than of HDL-2 or HDL_T_ on the release of VLDL surface lipids and exchangeable apolipoproteins during lipolysis. After lipolysis in the presence of HDL-2 and HDL_T_ we observed also the presence of pre-alpha mobility bands, which were not formed in the presence of HDL-3. These bands had a greater intensity when formed in the presence of HDL-2 than in the presence of HDL_T_. The presence of a pre-alpha mobility band can indicate that the HDL-2 subpopulation was not able to incorporate all the surface material released from VLDL during lipolysis under the experimental conditions used. It was previously reported that PL and FC, besides binding to HDL during VLDL lipolysis, can also bind to other acceptors, for example to albumin [30]. Furthermore, following lipolysis, the PL and apolipoproteins (e.g., apo E) released from VLDL were found in monolayer liposomes [31] or smaller particles [32].

The differences observed for VLDL surface material release during lipolysis and the remnant features can be related to the differences in the composition of the HDL subpopulations that affect VLDL lipolysis. Murdoch and Breckenridge showed that the release of lipids from VLDL during lipolysis seemed to be higher for HDL with a low FC/PL ratio [23]. HDL-3 is characterized by a significantly lower FC/PL ratio compared to HDL-2, as was observed in our study (Table 1) and others. Camont et al. showed that the FC content in HDL decreased with increasing particle density from 9.4% in HDL-2b to 5.3% in HDL-3c [33]. Moreover, the HDL-3 particles were also richer in phosphatidylcholine compared to the HDL-2 particles [33], which may result in an increased release of lipids from VLDL, since phosphatidylcholine-enriched particles can enhance the loss of lipids and apolipoproteins from VLDL during LPL-mediated lipolysis [29]. On the other hand, it was shown that HDL-2 is enriched in sphingomyelin, which increases the surface rigidity of the particles [34]. Sphingomyelin was reported to inhibit lipolysis by decreasing LPL affinity for TG and LPL activity [35], thereby reducing the release of the surface material and its delivery to HDL during lipolysis. Additionally, it was shown that sphingomyelin can decrease the binding capacity of apo E which may explain less release of apo E from VLDL during lipolysis in the presence of HDL-2 compared to HDL-3 [36].

Feng et al., assessing the impact of the HDL-C level on FC release during VLDL lipolysis showed that the release of FC from VLDL in the presence of HDL was U-shaped and the transfer of FC to HDL was most efficient at intermediate concentrations of HDL-C [20]. In our study, we examined the impact of HDL on VLDL during lipolysis for the constant VLDL-C:HDL-C cholesterol ratio; therefore, we were not able to assess the direct impact of HDL-C level on VLDL lipolysis. However, many previous studies have shown that subjects with low or high HDL-C levels had different HDL subpopulation profiles compared to those with intermediate HDL-C levels [37] and in subjects with high HDL-C, the larger and lighter HDL particles (HDL-2) predominated [37]. Thus, observed by us the differences in VLDL surface material release in the presence of HDL-2 and HDL-3, as well as between the subjects with high and low HDL-2/HDL-3 cholesterol ratios, may confirm that substantial changes in the HDL lipoprotein profile related to a significant increase of HDL-2 (and subsequently in an extremely high HDL-C level) may also result in disturbed the release of surface materials from VLDL, as was observed by Feng et al. [20,22]. 

The results of our study suggest that a condition of HDL-3 deficiency could be related to a significantly lower removal of apo E and FC from VLDL during lipolysis. Apo E is a major determinant of the binding and uptake of remnants by hepatocytes, but also of remnant endocytosis by subendothelial monocyte-derived macrophages. Thus, apo E-rich lipoprotein remnants can affect cholesterol accumulation and the formation of foam cells in artery walls, the hallmark cell of atherosclerotic plaque [38]. Moreover, it has been shown that abnormal lipoprotein catabolism related to hypertriglyceridemia can lead to the generation of modified, highly atherogenic electronegative LDL, which is characterized by a higher concentration of apo E, apo CIII and FC [39]. Chen et al. recently showed that apo E may play an important role in electronegative LDL-induced mitochondrial dysfunction in cardiomyocytes [40]. Further studies are needed to establish if the HDL disturbances related to the altered distribution of HDL subpopulations can affect VLDL catabolism and lead to the generation of modified apo E-rich LDL with increased atherogenic potential. Moreover, it would also be valuable to assess in future studies whether changes in the distribution of HDL subpopulations related to the pharmacological action; for example, an increase in the number of small HDL particles (corresponding to HDL-3) under the influence of fibrates and gemfibrozil [41,42] have a positive effect on the release of surface apolipoproteins and lipids from VLDL during lipolysis.

## 5. Conclusions

In conclusion, we showed that both HDL subpopulations are able to increase the efficiency of VLDL-TG lipolysis and significantly affect the release of VLDL surface remnants during lipolysis, but HDL-3 exhibited greater influence on this process. The results of our study confirm that HDL has a crucial role in the release of surface VLDL remnants during LPL-mediated lipolysis and indicate that alterations in the composition of HDL related to disturbances in the distribution of HDL-2 and HDL-3 can affect the features of VLDL remnants influencing atherosclerosis progression.

## Figures and Tables

**Table 1:** Lipid and apolipoprotein composition of VLDL, HDL_T_, and HDL-2 and HDL-3 subpopulations. Data are presented as mean ± SD or median with 25th and 75th percentiles (n = 10), * *p* < 0.05, ** *p* < 0.01, *** *p* < 0.001, compared to HDL-2.

Parameter	VLDL	HDL_T_	HDL-2	HDL-3
Triglycerides [mg/dL]	74.2 (52.1–110.3)	12.9 (10.9–14.9)	7.5 (5.4–10.7)	4.6 (4.2–4.9) *
Total cholesterol [mg/dL]	20.7 ± 12.4	46.8 ± 19.8	29.9 ± 15.3	16.7 ± 4.7 **
Free cholesterol [mg/dL]	6.8 ± 3.5	11.3 ± 6.0	7.2 ± 4.6	3.5 ± 1.3 *
Phospholipids [mg/dL]	25.6 ± 13.2	121.4 ± 40.7	74.4 ± 34.4	44.3 ± 9.1 *
Free cholesterol/Phospholipids ratio	0.265 ± 0.027	0.089 ± 0.021	0.093 ± 0.018	0.078 ± 0.013 ***
Apolipoprotein AI [mg/dL]	-	135.3 ± 40.2	82.0 ± 31.3	54.9 ± 9.8 *
Apolipoprotein B [mg/dL]	7.3 ± 3.2	-	-	-
Apolipoprotein CII [mg/dL]	1.7 ± 0.8	1.7 ± 0.2	1.0 ± 0.3	0.7 ± 0.1
Apolipoprotein CIII [mg/dL]	3.6 (2.7–4.5)	6.4 (4.6–8.3)	4.0 (2.6–5.9)	2.2 (1.9–3.2) *
Apolipoprotein E [mg/dL]	0.6 ± 0.2	0.6 ± 0.3	0.4 ± 0.3	0.2 ± 0.1 *

Data were analyzed by Paired *t*-test or Wilcoxon matched pairs test.

**Table 2:** Percentage [%] of surface lipids and apolipoproteins released from VLDL during lipolysis in the presence of HDL_T_ depending on the HDL-2 / HDL-3 cholesterol ratio. Value of 1.56 is a median of HDL-2 / HDL-3 cholesterol ratio calculated for study group. Data are presented as mean ± SD.

Parameter	HDL-2/HDL-3 Ratio <1.56 (n = 5)	HDL-2/HDL-3 Ratio ≥1.56 (n = 5)	*p*-Value
Free cholesterol [%]	27.1 ± 5.1	12.2 ± 5.2	0.002
Phospholipids [%]	47.7 ± 2.4	40.8 ± 6.1	0.044
Apolipoprotein E [%]	36.0 ± 13.9	19.5 ± 6.2	0.042
Apolipoprotein CII [%]	95.0 ± 5.0	88.2 ± 2.6	0.030
Apolipoprotein CIII [%]	90.6 ± 7.2	93.1 ± 4.6	0.529

Data were analyzed by Unpaired *t*-test. VLDL was incubated with LPL in the presence of HDL_T_ with the constant VLDL to total HDL cholesterol ratio of 1:1 in each experiment. The patients’ results were divided into group according to the basal HDL-2 / HDL-3 cholesterol ratio below or above 1.56.

## Data Availability

The data presented in this study are available on request from the corresponding author.
